# Evaluation of the efficacy of Biejia decoction pill combined with entecavir in the treatment of hepatitis B liver fibrosis/cirrhosis by VCTE

**DOI:** 10.1038/s41598-023-46459-4

**Published:** 2023-11-10

**Authors:** Lijun Wan, Chungen Hu, Fenfen Wang, Kedong Xu, Fan Li, Bo He, Zhengqiang Wu, Linfei Luo, Zhili Wen

**Affiliations:** Department of Gastroenterology Department, The Second Affiliated Hospital of Nanchang University, 1 Minde Road, Donghu District, Nanchang, Jiangxi China

**Keywords:** Hepatology, Diseases, Medical research

## Abstract

The vibration controlled transient elastography (VCTE) technique was used to assess the effectiveness of a Biejia Decoction pill in combination with Entecavir in the treatment of hepatitis B liver fibrosis/cirrhosis. We randomly selected 120 patients to receive entecavir and 119 patients to receive both entecavir and Biejia Decoction Pill, which both with hepatitis B liver fibrosis/cirrhosis visited the Second Affiliated Hospital of Nanchang University between January 2019 and February 2022. The observation group got ETV (entecavir) and Biejia Decoction pills, whereas the control group received only standard ETV antiviral medication. Based on the grading of the VCTE detection value (LSM) initially diagnosed for patients with hepatitis B liver fibrosis/cirrhosis, we divided the patients into two subgroups of liver fibrosis and cirrhosis. In addition, patients with liver fibrosis were divided into mild and moderate subgroups according to their VCTE values. Patients were measured for liver hardness after three, six, nine, and twelve months of treatment with VCTE. Biejia Decoction Pill combined with ETV on HBV liver fibrosis/cirrhosis was evaluated by comparing patients' changes in liver hardness and HBV-DNA negative conversion rates before and after treatment in each group at the same baseline. The LSM (liver elasticity value) of the observation group and the control group after treatment was lower than that before treatment, and the difference was statistically significant (P < 0.0001); The LSM of the observation group after treatment was significantly lower than that of the control group, and the difference was also statistically significant (P = 0.0005 < 0.05). In the subgroup of liver fibrosis, the number of patients with moderate and severe liver fibrosis who completely reversed liver fibrosis after treatment in the treatment group was far more than that in the control group, and the difference between the two groups was statistically significant (χ^2^ = 4.82 P = 0.028 < 0.05) 。 When the treatment course was more than 9 months, the negative conversion rate of patients in the observation group reached 87.4%, which was higher than that in the control group (70.8%), and the difference was statistically significant (P = 0.002 < 0.05); After 12 months of treatment, the negative conversion rate of patients in the observation group was as high as 95%, which was significantly higher than 76.67% in the control group (P < 0.001). The degree of liver fibrosis was significantly improved when Biejia Decoction Pill was combined with ETV in patients with liver fibrosis/cirrhosis due to hepatitis B. The virological response rate to HBV-DNA increased with the prolongation of treatment, and the Biejia Decoction Pill assists with entecavir in antiviral therapy.

## Introduction

In the world, over 1/3 of the population has had chronic hepatitis B infection or is currently infected, and about 12% of these people are HBsAg positive^1–5^.When effective treatment is not available or when patients refuse to receive it, 2% to 4% of patients develop liver fibrosis or even compensatory cirrhosis every year, and with the progression of the disease, patients with cirrhosis will gradually develop decompensated cirrhosis^6–8^.As a result of the severe complications of liver cirrhosis (portal hypertension, spontaneous peritonitis, hepatic encephalopathy, etc.), most patients are repeatedly hospitalized, and their daily lives are also adversely affected, and some severe patients even die from their liver cirrhosis^9–12^.There is no doubt that this is a great challenge for China's medical cause. In China, the incidence rate and prevalence of hepatitis B have decreased over the past few years since the vaccine policy was implemented, but this has not significantly reduced the incidence of end-stage liver disease or liver cancer caused by chronic hepatitis B in a short period of time^1,13^.Therefore, there is still a need to solve public health problems such as cirrhosis, end-stage liver disease, and even liver cancer caused by hepatitis B.

The liver biopsy is generally considered to be the gold standard for diagnosing liver fibrosis/cirrhosis, however, it is an invasive procedure that can cause pain, cannot be repeated, and has certain risks, which are often unacceptable to patients^14–17,23,24^.Furthermore, the quality of liver biopsy samples is generally restricted by factors such as sample size, sampling error, histological error, and inconspicuous histological findings at the early stages of fibrosis.Therefore, liver biopsy is not routinely used to detect liver fibrosis/cirrhosis^18–26^.

Thus, several noninvasive methods have been developed to evaluate liver fibrosis/cirrhosis due to the limitations of liver biopsy.In general, these methods are divided into biological ones based on the identification of serological markers and physical ones based on radiographic assessments of liver elasticity^27^.As serum markers' diagnostic accuracy is influenced by many factors, the diagnostic rate for early fibrosis is low, and there is literature showing that the variability of the natural evolution of HBV, immune activity, and inflammation will affect the accuracy of serum markers in diagnosing HBV fibrosis and cirrhosis. As a result, many patients with advanced fibrosis and cirrhosis are missed^28–31^.As a result of their limitations, serum markers that indicate liver fibrosis/cirrhosis are not used widely for hepatitis B liver fibrosis/cirrhosis^30–34^.

An elastic image reflects the mechanical properties of tissues and their hardness, and fibrosis levels are positively correlated with hardness^35^.Elastography imaging methods most commonly used are vibration controlled transient elastography (VCTE), 2D shear wave elastography (2D SWE), point shear wave elastography (pSWE) and magnetic resonance elastography (MRE)^36,37^.American Association of Gastroenterology (ACG), European Association for Liver Research (EASL), and European Federation of Medical and Biological Ultrasound Societies (EUROSON) have recently recommended VCTE for the evaluation of chronic liver disease (CLD) and fibrosis associated with chronic viral hepatitis^38,39^.According to EUROSON, VCTE (also known as FibroScan, Echosens) greater than 7.6 kPa indicates significant fibrosis (F2), while 11.0–13.6 kPa indicates cirrhosis (F4).The literature suggests that although VCTE cannot replace liver biopsy, it can be used as a substitute for patients who refuse liver biopsy and as a method of evaluating CLD over a long period of time^38–40^.

There was a time when people believed liver fibrosis was irreversible, especially when the disease progressed to the stage of cirrhosis.In recent years, numerous studies have demonstrated that liver fibrosis is a reversible pathophysiological condition^39,40^.Currently, antiviral treatment is the main treatment for hepatitis B liver fibrosis.The treatment can slightly reverse liver fibrosis, but it is limited by drug resistance and poor virological responses in some patients.Despite long-term antiviral treatment, some patients may develop liver fibrosis^41^.There has been extensive research showing that traditional Chinese medicine can effectively treat liver fibrosis and improve liver function in recent years^42^.Biejia Decoction pill, a traditional Chinese medicine compound, significantly improved liver fibrosis in vivo and in vitro.A Biejia Decoction pill was used to inhibit the proliferation and decrease collagen content of activated HSC-LX-2 cells, while inhibiting collagen deposition in these cells, as reported by the researchers.The researchers also found that Biejia Decoction pill down-regulated TGF-β 1 and Smad3 expression and changed the percentage of G0/G1 and S phase cells, suggesting that Biejia Decoction pill may work by preventing liver injury and reversing liver fibrosis as its primary mechanism^43,44^.Additionally, relevant pharmacological studies have demonstrated that Biejia Decoction pill can stimulate the degradation of collagen tissue, as well as dissolve and absorb liver fibrosis, reduce collagen synthesis, decrease excessive deposition, and effectively reverse liver fibrosis^45–47^.

This paper attempts to evaluate and identify the changes of liver elasticity value of patients with liver fibrosis and cirrhosis caused by chronic hepatitis B before and after treatment using VCTE (liver fibrosis scanning), and to quantitatively evaluate the efficacy of ETV and Biejia Decoction Pill when treating liver fibrosis and cirrhosis in hepatitis B patients.So as to provide more clinical data for the treatment plan and clinical management of chronic hepatitis B patients with Biejia Decoction Pill's effect on liver fibrosis.

## Materials and methods

### Research object

#### Research object and grouping

**Figure 1:**
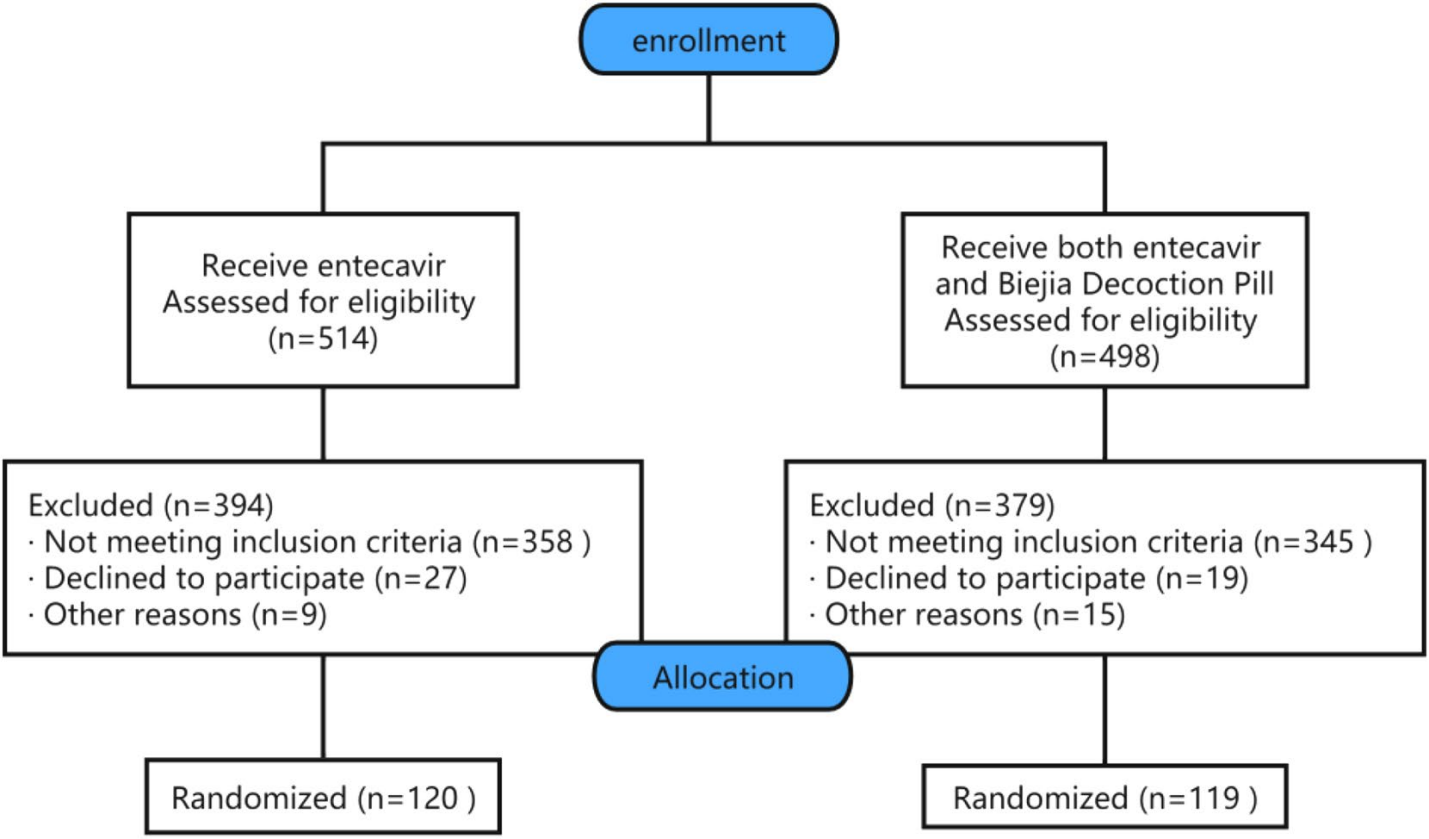
CONSORT flow diagram.

We retrospectively selected 120 patients to receive entecavir and 119 patients to receive both entecavir and Biejia Decoction Pill at the same time (informed and agreed by the patients), which both with hepatitis B liver fibrosis/cirrhosis visited the Second Affiliated Hospital of Nanchang University between January 2019 and February 2022 randomly (aged 19–83 years, male to female ratio of about 2:1 ).These patient information and data were obtained from the hospital network database.An observation group was treated with entecavir capsules (Fujian Guangshengtang Pharmaceutical Co., Ltd,GYZZ H20110172,0.5 mg/qd) combined with a Biejia Decoction Pill^*^ (Sinopharm Group Zhonglian Pharmaceutical Co., Ltd,GYZZ Z42020772, 3g/tid) . A control group was treated with entecavir capsules (0.5 mg/qd) alone. (CONSORT Flow Diagram in Fig. 1).

*Biejia Decoction Pill:comprised of 23 herbal medicines, including Biejiajiao (Trionycis Carapax), Ejiao (Colla Corii Asini), Fengfang (Vespae Nidus), Shufuchong (Armadillidium), Chaihu (Bupleuri Radix), Tubiechong (Eupolyphaga Steleophaga), Qianglang (Liinnaeus), Xiaoshi (Saltpeter), Huangqin (Scutellariae radix), Banxia (Pinelliae Rhizoma), Dangshen (Codonopsis radix), Ganjiang (Zingiberis Rhizoma), Houpo (Magnoliae Officinalis cortex), Guizhi (Cinnamomi Ramulus), Shegan (Belamcandae Rhizome), Baishao (Paeoniae Radix Alba), Mudanpi (Moutan Cortex), Dahuang (Rhei Radix et Rhizoma), Lingxiaohua (Campsis Flos), Tinglizi (Descurainiae Semen),Taoren (Persicae Semen), Shiwei (Pyrrosiae Folium), and Qumai (Dianthi Herba).

#### Inclusion criteria

1. Improve the detection of hepatitis B, HBV-DNA, and other examinations, and confirm the diagnosis of hepatitis B infected patients;
2. Diagnostics of liver fibrosis and cirrhosis based on the VCTE examination^48^;
3. Entecavir is taken alone or in combination with Biejia Decoction Pill without taking other drugs that affect the liver function [this study was approved by the Ethics Committee of the Second Affiliated Hospital of Nanchang University (approval #20190913). All subjects provided written informed consent].

#### Exclusion criteria

1. Other viral infections (such as hepatitis A, hepatitis C, and hepatitis D) were excluded;
2. Those with alcohol, nonalcoholic, drug-induced, genetic metabolic, and autoimmune liver diseases were excluded;
3. We exclude patients with malignant tumors, congenital or acquired immunodeficiency, use of immunosuppressants, and patients over 18 years old, pregnant or lactating women, and those with cardiac or cerebrovascular complications;
4. VCTE was excluded from patients with obesity, abnormal bilirubin levels, and other factors affecting its accuracy and efficacy.

#### Observation indicators and evaluation criteria

1. Liver hardness value: The FibroScan detector is used to measure liver elasticity (LSM) .
2. The level of HBV-DNA was detected by PCR (polymerase chain reaction) before and after treatment.Every three months, HNB-DNA levels were rechecked for all patients.

#### Adverse reaction

The main adverse reactions of the included patients were gastrointestinal discomfort, skin allergy, headache, etc.

### Research method

#### General information

Baseline data in general: a variety of general information about the selected subjects, such as age, sex, history of drinking or smoking, and so forth, was recorded.

FibroScan detection: in elastic imaging, VCTE is commonly referred to as fiber scanning.To improve the detection success rate, all measurements are tested by experienced and professional physical examination doctors.Software records successful measurements without recording unsuccessful ones.It should contain at least 10 effective measurement values, with a success rate of 60% (the ratio of effective measurement values to total measurement values).This value is calculated by taking the range of quartiles (the variability of measurement value) and adding 30% to the median (VCTE grading criteria for liver fibrosis are listed in Table 1 below).

**Table 1:** FibroScan examination of liver fibrosis grading in our hospital.

LSM (kPa)	[7.3, 9.7)	^[9.7, 12.4)^	^[ 12.4, 17.5)^	≥ 17.5
Grading of liver fibrosis	Mild	Moderate	Severe	Cirrhosis

#### Statistical methods

Continuous variables of general data conform to normal distribution and are expressed as mean ± standard deviation; a non-normal distribution is expressed as a median (quartile) [M (P25–P75)];Frequency and percentage of total number (n%) are used to express classification variables and count variables.For intergroup continuous variables that conform to the normal distribution and have homogeneous variance, the independent sample t-test is used, and for variance that is uneven or does not conform to the normal distribution, the Mann–Whitney test is used; an intra-group comparison was conducted using a paired sample t-test;A chi-square test or Fisher's exact test was used to compare classification and counting data.P < 0.05 is considered statistically significant.The data were analyzed statistically using SPSS version 21.0 (IBM Corp, Armonk, NY).

### Approval of the research protocol & informed consent

The study was approved by the Ethics Committees of The Second Affiliated Hospital of Nanchang University (approval #20190318).

## Result

### Comparison of general clinical data

This study included 239 patients, including 119 observational patients and 120 control patients;In terms of men and women, gender, age, BMI (body mass index), smoking, drinking, AST (aspartate aminotransferase), TC (total cholesterol), TG (triglycerides), TBIL (total bilirubin), VCTE-CAP (fibroscan liver steatosis detection value), HBV-DNA (hepatitis B virus load), and treatment course (P > 0.05), there was no significant difference between the observation group and the control group. (Details can be found in Table 2).

**Table 2:** Comparison of general information.

Variable	Group	
	Control group	Observation group
Patients (people)	120	119*
Gender, male , n (%)	79 (64.70%)	81 (67.44%)*
Age (years)	44 (34, 53.75)	43.5 (32, 53)*
BMI (kg/m^2^)	21.21 ± 3.78	21.73 ± 3.82*
Smoking history, n (%)	57	57*
Drinking history, n (%)	25	28*
ALT (U/L)	65.7 (31. 1, 124.7)	68.2 (32.4, 135.7)*
AST (U/L)	58.3 (32.6, 91.5)	57.9 (31.8, 90.7)*
TBIL (μmol/L)	14.3 (9.67, 18.54)	14.5 (8.78, 18.6)*
TC (mmol/L)	5.05 ± 1.03	5. 11 ± 1. 15*
TG (mmol/L)	1.79 ± 1.01	1.81 ± 1. 11*
VCTE-CAP (dB/m )	190 (172,209)	193 ( 176,210)*
HBV-DNA (IU/mL)	4263.47 ± 407.26	4264.58 ± 408.08*
Course (months)	6 (6, 12)	6 (6, 12)*

*P > 0.05.

### A comparison of the curative effects of observation and control groups

After treatment, the liver hardness values of the observation group and control group are lower than those before treatment. The difference is statistically significant (P < 0.0001).

**Figure 2:**
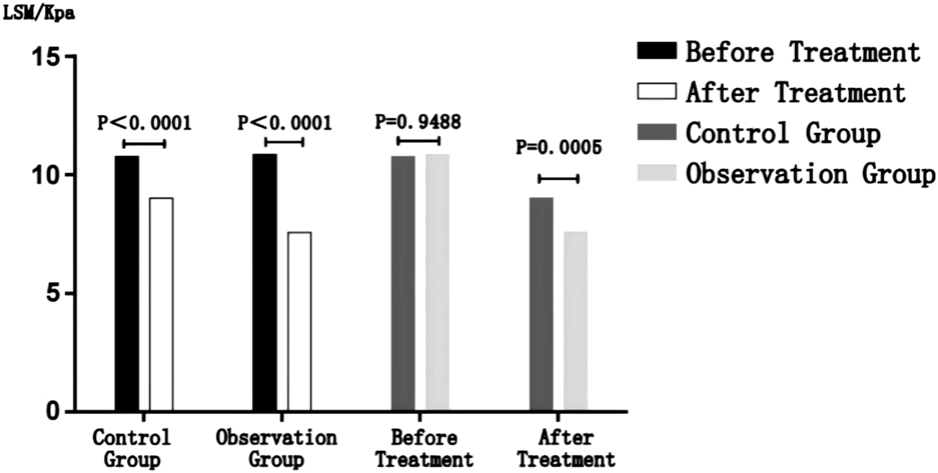
Efficacy comparison.

There was no statistically significant difference between the liver hardness values of the observation and control groups before treatment (P = 0.9488 > 0.05); After treatment, the liver hardness value of the observation group is lower than that of the control group, and the difference is statistically significant (P = 0.0005 < 0.05) (details can be found in Fig. 2 and Table 3).

**Table 3:** Efficacy comparison.

Group	Before treatment LSM/kPa	After treatment LSM/kPa	*P*
Control group	10.75 (8.8, 17.8)	9 (7. 1, 16.25)	< 0.0001
Observation group	10.85 (8.8, 18)	7.55 (5.8, 12.43)	< 0.0001
*P*	0.9488	0.0005	

### Hepatic fibrosis and cirrhosis subgroups: comparison of curative effects

#### Comparison of general data

The two groups of patients were divided based on the degree of fibrosis into hepatic fibrosis (171 cases) and cirrhosis (68 cases).In the hepatic fibrosis group, the VCTE detection range is [7.3 kPa, 17.5 kPa); in the liver cirrhosis group, it is ≥ 17.5 kPa.In both subgroups, there is no significant difference in gender, age, IBM, smoking, drinking, AST, TC, TG, TBIL, VCTE-CAP, etc. between the control and observation groups (*P* > 0.05).Hepatic fibrosis subgroup results showed no significant difference between observation and control groups for ALT (*P* > 0.05); An observation group with liver cirrhosis had a significantly higher ALT than a control group, and this difference was statistically significant (*P* < 0.05) (details can be found in Table 4).

**Table 4:** Comparison of general information *P < 0.05 (comparison between control group and observation group).

Variable	Subgroup			
	Liver fibrosis subgroup		Liver cirrhosis subgroup	
	Control group	Observation group	Control group	Observation group
Patients (people)	85	86	35	33
Gender, male , n (%)	55 (64.71%)	58 (67.44%)	24 (68.57%)	23 (69.70%)
Age (years)	44 (33.00, 51.50)	41.5 (31.00,51.50)	45 (36.00, 65.00)	44 (37.55,58.25)
BMI (kg/m^2^)	20.21 ± 3.65	21.43 ± 3.52	21.32 (19.36, 24.30)	21.13 (20.15, 24.90)
Smoking history, n (%)	39 (45.88%)	38 (44.19%)	18 (51.43%)	19 (57.58%)
^Drinking history, n (%)^	19 (22.35%)	21 (24.42%)	6 (17.14%)	7 (21.21%)
ALT (U/L)	59.82 (21.13, 124.72)	65.44 (25.41, 135.73)	49.17 (29.50, 76.73)	61.89 (40.62, 98.50)*
AST (U/L)	45.42 (30.58, 85.50)	46.50 (29.84, 86.88)	59.94 (35.69, 79.56)	58.21 (33.85, 83.49)
TBIL (μmol/L)	14.32 (9.67, 18.54)	14.49 (8.78, 18.60)	15.42 (13.33, 20.90)	16.00 (14.63, 21.30)
TC (mmol/L)	5.10 ± 0.98	5.15 ± 1.13	4.98 ± 1.18	5.05 ± 1.21
TG (mmol/L)	1.75 ± 1.10	1.78 ± 1.21	1.81 ± 0.98	1.83 ± 1.03
VCTE-CAP (dB/m)	185 (169, 199)	189 (170, 208)	196 (181, 218)	199 (187, 216)

#### Before and after treatment comparison of the control group in each subgroup

**Figure 3:**
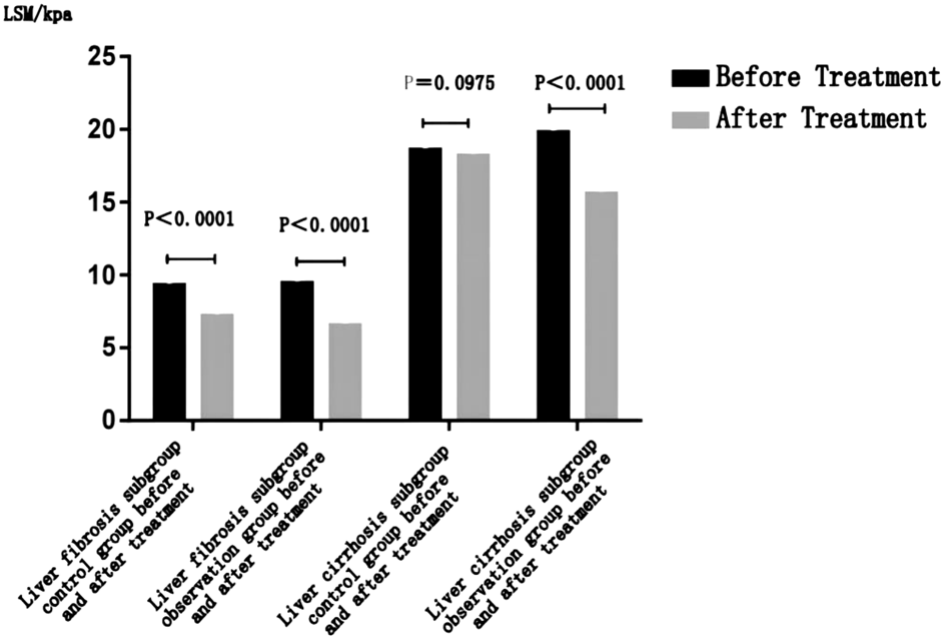
Comparison of curative effects of each subgroup before and after treatment.

In the liver fibrosis subgroup, the liver stiffness value after treatment in the control group was lower than that before treatment, and the difference was statistically significant (*P* < 0.0001); in the subgroup of liver cirrhosis, there was no significant difference in liver hardness before and after treatment in the control group (*P*** = **0.0975 > 0.05) (details can be found in Fig. 3 and Table 5).

**Table 5:** Comparison of curative effects between liver fibrosis subgroup and cirrhosis subgroup.

Subgroup		Before treatment LSM/kPa	After treatment LSM/kPa	Curative effects	*P*
Liver fibrosis subgroup	Control group (85 )	9.4 (8.3, 11.7)	7.3 (6.85, 9.4)	− 1.5 ( −2.15 , −0.09)	< 0.0001
	Observation group (86 )	9.55 (8.4, 11.5)	6.65 (5.4, 7.925)	−3 ( −3.85, −2.00)	< 0.0001
	*P*	0.8466	< 0.0001	< 0.0001	
Liver cirrhosis subgroup	Control group (35 )	18.7 (18, 25.8)	18.3 (16.3 , 28.4)	− 1.5 (−2.2 , 1.1)	0.0975
	Observation group (33)	19.9 (18, 26.1)	15.7 (13.1, 19.7)	−5.7 (−7.2, −4.1)	< 0.0001
	*P*	0.7047	< 0.0001	< 0.0001	

#### Before and after treatment comparison of the observation group in each subgroup

No matter in the liver fibrosis group or cirrhosis subgroup, the liver hardness value of the observation group after treatment is lower than that before treatment, and the difference between the two groups is statistically significant (*P* < 0.0001) (details can be found in Fig. 3 and Table 5).

#### Observation group and control group after treatment: comparison of curative effects in each subgroup

**Figure 4:**
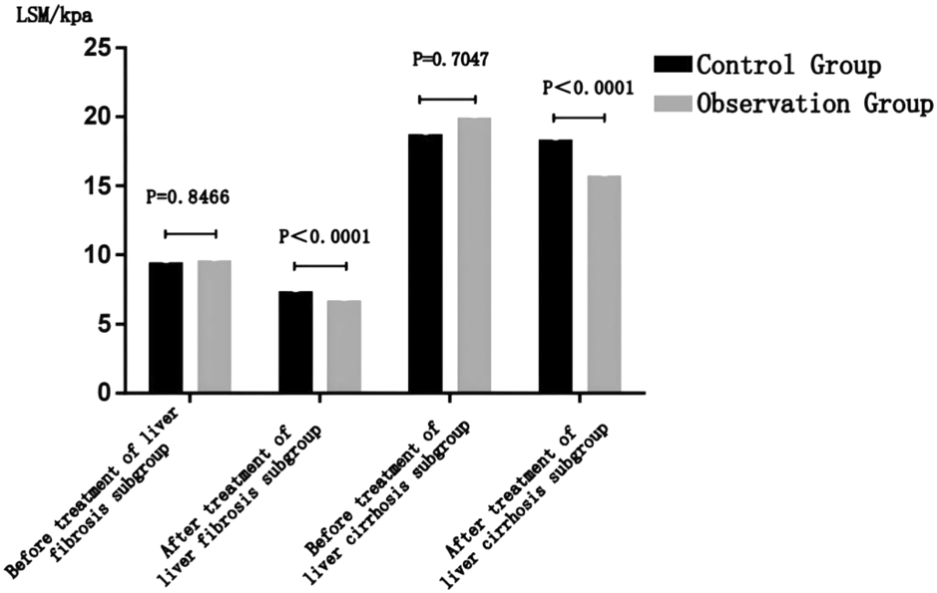
Observation group and control group after treatment: comparison of curative effects in each subgroup.

Before treatment, the liver hardness value of the observation group of each subgroup was higher than that of the control group, but there was no significant statistical difference (*P* > 0.05); after treatment, the liver hardness of the observation group of each subgroup was lower than that of the control group, and the difference was statistically significant (*P* < 0.0001) (details can be found in Fig. 4 and Table 5).

### A comparison of the curative effects of hepatic fibrosis in mild and moderate-severe subgroups

#### Comparison of general data

Based on the VCTE detection value of patients, hepatic fibrosis patients were divided into mild and moderate-severe subgroups (patients with detection values [7.3,9.7) kPa were mild liver fibrosis, whereas patients with detection values [9.7, 17.5) kPa were moderate-severe).In terms of gender, age, AST, and other variables, there was no significant statistical difference between the two subgroups (*P* > 0.05). In the observation group of the mild fibrosis subgroup, ALT levels were higher than in the control group, but the difference was not statistically significant (P > 0.05); in the observation group of moderate-severe fibrosis subgroups, ALT levels were also higher than those in the control group, and the difference was statistically significant (P < 0.05) (details can be found in Table 6).

**Table 6:** General data comparison of Liver fibrosis subgroup.

Variable	Subgroup			
	Mild fibrosis subgroup		Moderate–severe fibrosis subgroup	
	Control group	Observation group	Control group	Observation group
Patients (people)	44	47	41	39
Gender, male, n (%)	25 (56.82%)	30 (63.83%)	30 (73.17%)	28 (71.79%)
Age (years)	41.93 ± 10.93	40.34 ± 12.77	46.24 ± 13.51	46.10 ± 14.49
Smoking history, n (%)	14 (31.82%)	15 (31.91%)	25 (60.98%)	24 (61.54%)
Drinking history, n (%)	15 (34.09%)	19 (42.43%)	24 (58.54%)	20 (51.28%)
ALT (U/L)	58.40 (20.50, 122.93)	63.95 (34.31, 133.72)	61.22 (24.00, 134.40)	79.45 (40.21, 146.23)*
AST (U/L)	41.62 (29.64, 79.71)	42.60 (28.81, 81.00)	48.13 (30.62, 98.00)	49.32 (31.71,99.58)

*P < 0.05 (comparison between control group and observation group).

#### A comparison of the curative effects between the two subgroups

**Figure 5:**
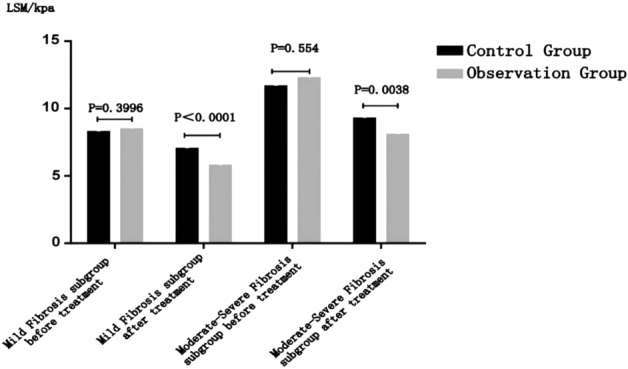
Comparison of curative effects between mild fibrosis subgroup and moderate-severe fibrosis subgroup.

Before treatment, the liver hardness values in the observation group of the two subgroups were greater than those in the control group, but there was no significant statistical difference (P > 0.05); after treatment, the liver hardness of the observation group in the two subgroups was significantly lower than that of the control group, and the difference was statistically significant (P < 0.05) (details can be found in Fig. 5 and Table 7).

**Table 7:** Comparison of curative effects between mild fibrosis subgroup and moderate-severe fibrosis subgroup.

Degree of liver fibrosis		Before treatment LSM/kPa	After treatment LSM/kPa	Effective treatment*, n (n%)
Mild	Control group (44)	8.3 (7.625, 8.9)	7.05 (6.6, 7.3)	31 (70.45%)
	Observation group (47)	8.5 (7.9, 9)	5.8 (5.2, 6.9)	36 (76.6%)
	χ^2^	NA	NA	0.441
	*P*	0.3996	< 0.0001	0.506
Moderate–severe	Control group (41)	11.7 (10.6, 13.35)	9.3 (8. 1,10.75)	6 (14.63%)
	Observation group (39)	12.3 (10.5, 13.8)	8.1 (6.8,9.4)	14 (35.9%)
	χ^2^	NA	NA	4.82
	*P*	0.554	0.0038	0.028

*Effective treatment: VCTE detection value < 7.3 kPa after treatment.

**Figure 6:**
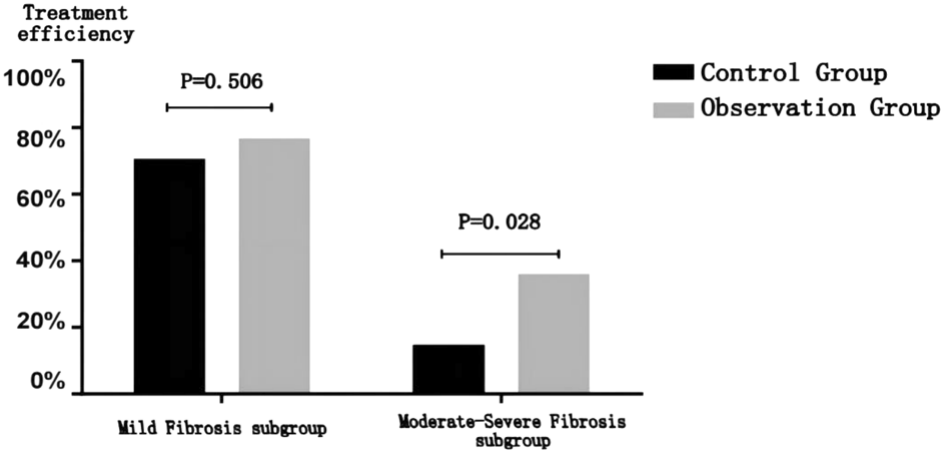
Comparison of the curative effects between the two subgroups.

Compare the situation of complete reversal of liver fibrosis (VCTE detection value < 7.3 kPa after treatment) between the two subgroups: a statistically insignificant difference was found between the observation and control groups in the mild fibrosis subgroup with an effective rate of treatment of 76.6% in the observation group and 70.45% in the control group (χ^2^ = 0.441 *P* = 0.506 > 0.05) ; an observation group with moderate-severe liver fibrosis had a 35.9% effective rate, higher than a control group with 14.63%, and the difference was statistically significant between the two groups (χ 2 = 4.82 *P* = 0.028 < 0.05) (details can be found in Fig. 6 and Table 7).

### Comparing the rate of HBV-DNA turning negative between observation and control groups

**Figure 7:**
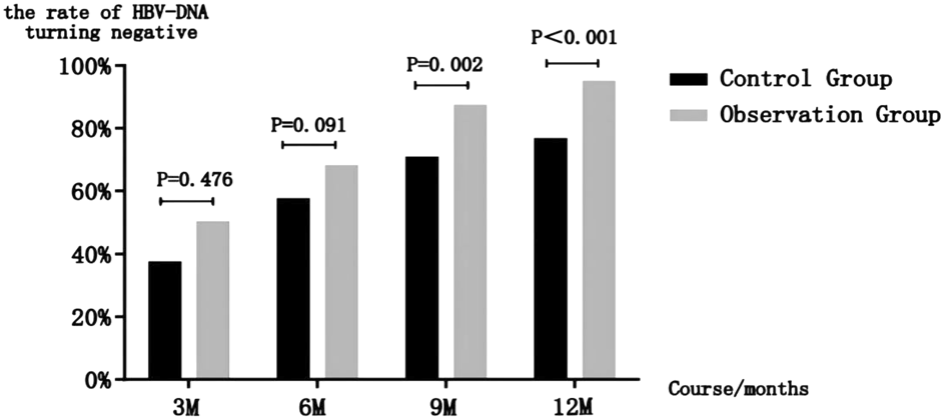
Comparing the rate of HBV-DNA turning negative between observation and control groups n (n %).

An observation group with 3–6 months of treatment has a slightly higher HBV-DNA negative rate than a control group, but there is no statistically significant difference between the two groups (*P* > 0.05); after more than 9 months of treatment (including 9 months), the observation group's HBV-DNA negative rate reached 87.4%, significantly higher than the control group's 70.8%,and the difference was statistically significant (*P* = 0.002 < 0.05) ; it takes more than 12 months (including 12 months), and the rate of HBV-DNA negative conversion in the observation group is as high as 95%, which is significantly higher than in the control group (76.67%), and the difference is statistically significant (P < 0.001) (details can be found in Table 8) (Fig. 7).

**Table 8:** Comparing the rate of HBV-DNA turning negative between observation and control groups n (n %).

Course (months)	3	6	9	12
Observation group (119)	50 (42.02%)	81 (68. 07%)	104 (87.39%)	113 (94.96%)
Control group (120)	45 (37.50%)	69 (57.50%)	85 (70.83%)	92 (76.67%)
χ2	0.509	2.855	9.906	16.383
*P*	0.476	0.091	0.002	< 0.001

### Adverse reactions were compared

In 119 cases, gastrointestinal discomfort and skin allergy occurred in two cases; In the control group, there were two cases of gastrointestinal discomfort and one case of headache. The adverse reaction rate of observation group and control group were 3.36% and 2.50%, respectively (P > 0.05). There was no statistical significance. No other serious adverse reactions were observed.

## Discussion

A lack of effective and safe therapeutic options for chronic hepatitis B makes it difficult to clear latent virus-like particles from the body, and clinicians mainly use antiviral therapy to control the disease's progression.Even so, in some patients, hepatitis B virus replication is not active, so it would not cause serious acute liver problems.But there's a possibility that hepatitis will eventually turn into liver fibrosis, which worsens liver function, and eventually leads to cirrhosis with end-stage liver disease, and hepatocellular carcinoma.

According to a longitudinal analysis of untreated hepatitis B patients, they have a cumulative risk of developing cirrhosis of up to 20% within five years. In contrast to patients with cirrhosis caused by other causes, patients with this cause have significantly lower survival rates and prognoses, with a five-year survival rate of only 15% to 40%^1^.There are still some patients with hepatitis B whose disease process cannot be effectively controlled even after receiving active antiviral treatment in time, causing them to develop cirrhosis or even end-stage liver disease, which may be caused by chronic hepatitis B low-level viremia (LLV) during the treatment cycle.

LLV refers to a persistent or intermittent HBV DNA load greater than the lower limit of detection, but not exceeding 2000 IU/ml.An analysis of risk factors for fibrosis progression in patients with persistently low levels of hepatitis B virus load during treatment shows that hypoviremia affects all stages of hepatitis B, and these patients have a higher risk of liver fibrosis than those with maintained virological response (MVR) .According to the study, there was a 22 percent rate of liver fibrosis progression during antiviral treatment, a 24 percent rate of uncertain liver fibrosis progression, and a 117 percent rate of liver fibrosis regression among 163 patients with obvious liver fibrosis.COX univariate and multivariate analysis of the risk factors for the progression of liver fibrosis showed that the risk of further aggravation of liver fibrosis in LLV patients increased nearly five times when the course of treatment was 7 to 8 weeks^49^. It has been reported that even after active antiviral treatment, 1/5–3/5 patients still suffer from disease progression after two years, and in severe cases, even liver failure.After antiviral treatment, one third of the patients developed LLV, which may explain their poor prognosis.According to the study, patients without a viral response are more likely to be stimulated by various cytokines to produce a series of immune responses during liver fibrosis, which is one of the major factors contributing to liver cancer progression.Accordingly, LLV patients are more likely than MRV patients to develop liver cancer^50^.

In this study, a small number of patients in each subgroup experienced fibrosis aggravation during the treatment stage, but the aggravation degree in the observation group was much lower than it was in the control group, which might be related to the enhancement of anti-virus effects of entecavir by Biejia Decoction Pills.After the treatment with Biejia Decoction Pills, with the prolongation of the course of treatment, when the course of treatment ≥ 9 months, the rate of HBV-DNA turning negative in the observation group reached 87.4%. This number was significantly higher than that of the control group (70.8%), and the difference was statistically significant (P = 0.002 < 0.05).The rate of HBV-DNA turning negative in the observation group can reach 95% when the treatment period is ≥ 12 months.Even some of the patients with liver cirrhosis experienced cirrhosis reversal after one year of combined treatment (LSM < 17.5).

Several studies have shown that liver fibrosis continues to progress in some patients with MRV, but the specific mechanism remains unclear.This may be related to metabolic syndrome, alcohol consumption, and other factors.There was still improvement of liver fibrosis in some patients with MRV in the observation group of this study, suggesting that Biejia Decoction Pills could reverse liver fibrosis not only by strengthening antiviral treatment with entecavir.Using Biejia Decoction Pills, modern pharmacological research shows that it clears the liver, reduces liver fibrosis, inhibits collagen production, and decreases serum transaminase levels, inhibits the release of inflammatory factors, and reduces liver fibrosis by reducing liver inflammatory activity^51^.

Based on a domestic study observing the effect of Biejia Decoction Pills on the improvement of liver inflammation and fibrosis in mice models of liver fibrosis, it was found that Biejia had obvious inhibitory effects on mononuclear macrophages through mouse experiments, which theoretically proved that Biejia was anti-inflammatory and anti-fibrotic.Based on these results, its mechanism of action may be related to inhibition of monocyte infiltration and reduction of monocyte pro-inflammatory and pro-fibrotic cytokine secretion^52^.The study confirmed the possibility of treating liver fibrosis with monocytes as a target, as well as provided new ideas on how to clarify liver fibrosis at the molecular level in the future.

A further subgroup analysis of patients in the liver fibrosis group showed no significant difference in reversing liver fibrosis between the observation group and the control group in the mild fibrosis subgroup (χ2 = 0.441 *P* = 0.506 > 0.05); only three (6.5%) patients in the observation group experienced fibrosis aggravation compared to 8 (18.2%) patients in the control group.The reversal rate of the observation group was significantly higher than that of the control group in the moderate and severe fibrosis subgroups, and the difference was statistically significant (χ2 = 4.82 *P* = 0.028 < 0.05).Therefore, for patients with early mild liver fibrosis and MRV, only entecavir can be given long-term antiviral treatment, and monitor LSM regularly.The risk of liver fibrosis progression is high in patients with LLV, so we can adjust antiviral drugs for patients with poor or no response to virology and use compound Biejia decoction pills (long-term treatment is recommended) toachieve viral response and reverse liver fibrosis as soon as possible.

Nearly 20% of patients with mild liver fibrosis have slight aggravation.In such patients, it is essential to undergo regular VCTE examinations to monitor the progression of liver fibrosis, as well as to monitor HBV-DNA levels in order to understand the virus' replication status.According to the study, more patients with moderate and severe fibrosis and cirrhosis developed fibrosis aggravation after antiviral treatment, highlighting the poor antiviral and fibrosis reversal efficacy of entecavir.Therefore, no matter whether such patients have MRV or not, they should add Compound Biejia Decoction pill in time to control the progress of liver fibrosis, and adjust the course of treatment according to the results of HBV-DNA, VCTE and other tests, so as to delay the progression of liver fibrosis to the greatest extent possible.

To sum up, for patients with chronic hepatitis B, on the basis of entecavir capsule treatment, supplemented with Biejia Decoction Pill treatment will help to control the further development of liver fibrosis in patients, and may even reverse liver fibrosis and improve clinical symptoms.In addition, patients had a higher response rate to the HBV DNA virus when the use time of Biejia decoction pills was prolonged, which contributed to the efficacy of the entecavir antiviral treatment.In recent years, some domestic studies have shown that traditional Chinese medicine can reverse chronic liver fibrosis caused by a variety of factors. In addition to being cheap, safe, and easy to access, these drugs provide more hope in controlling liver fibrosis and cirrhosis in the future^53,54^. Next, we should conduct a systematic study on the pathology and molecular level of these traditional Chinese medicines that have been proven to have a definite effect on liver fibrosis, to clarify the cellular and molecular mechanisms of fibrosis regression, and to investigate the possible pathogenesis of liver fibrosis in order to develop new drugs.

## Conclusion

Hepatitis B liver fibrosis/cirrhosis patients treated with Biejia Decoction Pills and ETV showed significant improvement in liver fibrosis;The virological response rate of HBV-DNA increased with prolonged treatment, suggesting that Biejia Decoction Pillsmay help entecavir in its antiviral activity.

## Data Availability

The datasets are available by request to the corresponding author.
